# Two-Component Signal Transduction Systems of Pathogenic Bacteria As Targets for Antimicrobial Therapy: An Overview

**DOI:** 10.3389/fmicb.2017.01878

**Published:** 2017-10-10

**Authors:** Sandeep Tiwari, Syed B. Jamal, Syed S. Hassan, Paulo V. S. D. Carvalho, Sintia Almeida, Debmalya Barh, Preetam Ghosh, Artur Silva, Thiago L. P. Castro, Vasco Azevedo

**Affiliations:** ^1^Laboratório de Genética Celular e Molecular, Departamento de Biologia Geral, Instituto de Ciências Biológicas (ICB), Universidade Federal de Minas Gerais, Belo Horizonte, Brazil; ^2^Biochemistry Group, Department of Chemistry, Islamia College University, Peshawar, Pakistan; ^3^Centre for Genomics and Applied Gene Technology, Institute of Integrative Omics and Applied Biotechnology, Purba Medinipur, India; ^4^Department of Computer Science, Virginia Commonwealth University, Richmond, VA, United States; ^5^Instituto de Ciências Biológicas, Universidade Federal do Pará, Belém, Brazil; ^6^Instituto de Ciências da Saúde, Universidade Federal da Bahia, Salvador, Brazil

**Keywords:** bacterial two-component signal transduction system, virulence and antibiotic resistance, inhibitors for kinases and response regulators

## Abstract

The bacterial communities in a wide range of environmental niches sense and respond to numerous external stimuli for their survival. Primarily, a source they require to follow up this communication is the two-component signal transduction system (TCS), which typically comprises a sensor Histidine kinase for receiving external input signals and a response regulator that conveys a proper change in the bacterial cell physiology. For numerous reasons, TCSs have ascended as convincing targets for antibacterial drug design. Several studies have shown that TCSs are essential for the coordinated expression of virulence factors and, in some cases, for bacterial viability and growth. It has also been reported that the expression of antibiotic resistance determinants may be regulated by some TCSs. In addition, as a mode of signal transduction, phosphorylation of histidine in bacteria differs from normal serine/threonine and tyrosine phosphorylation in higher eukaryotes. Several studies have shown the molecular mechanisms by which TCSs regulate virulence and antibiotic resistance in pathogenic bacteria. In this review, we list some of the characteristics of the bacterial TCSs and their involvement in virulence and antibiotic resistance. Furthermore, this review lists and discusses inhibitors that have been reported to target TCSs in pathogenic bacteria.

## Introduction

Microbes are the most versatile living organisms on the planet. They can be found in environments where plants and animals cannot survive, such as in hydrothermal vents on the ocean floor or glaciers. Bacteria have developed a number of features (e.g., signal transduction systems) that allow crosstalk between the intracellular and extracellular environments. The bacterial repertoire for sensing environmental conditions includes regulatory proteins that bind to secondary metabolites or ions, resulting in increased affinity for specific regulatory DNA sequences. Examples of well-studied regulatory proteins include the Catabolite Regulatory Protein (CRP, which senses cyclic adenosine monophosphate accumulated under low availability of glucose) and Ferric Uptake Regulator (Fur, which binds to Fe^2+^) (Nixon et al., 1986; Hoch, 2000; Stock et al., 2000). The bacterial capability of responding to external stimuli is conferred by a specialized signal transduction mechanism, which relies on the two-component systems (TCSs) (Cheung and Hendrickson, 2010; Groisman, 2016). In general, a TCS comprises a sensor protein (histidine kinase or HK) and its corresponding response regulator (RR). Usually, as each particular TCS is specialized to respond to a specific environmental signal (e.g., pH, nutrient levels, osmotic pressure, redox state, quorum-sensing proteins and antibiotics), multiple TCSs may be present in a single bacterial cell.

The virulence factors of bacterial pathogens and the regulatory systems that monitor their expression are predominantly attractive for therapeutic intervention. Recently, TCSs have emerged as potential targets for antibacterial drug design for a number of reasons. As a common practice, conventional antibiotics target specific bacterial proteins that perform essential functions in the pathogen; however, a drug that targets TCSs could be highly effective because it impairs upstream regulatory functions related to the pathogen’s physiology instead of affecting only specific downstream functions. Thus, the use of TCSs for drug development provides an alternative strategy for combating microbial infections, including those caused by pathogens resistant to currently available antibiotics (Gotoh et al., 2010). In addition, as the expression of many antibiotic resistance determinants is regulated by TCSs (Poole, 2012), an effective antimicrobial treatment could also be achieved by combining conventional and TCS-directed drugs. Still, selective TCS inhibitors are supposed to have the least toxicity to mammalian cells, since the bacterial signal transduction systems based on the phosphorylation of histidine differ from the serine/threonine and tyrosine phosphorylation signaling systems normally found in higher eukaryotes. Finally, a high degree of structural homology between the catalytic domains of the histidine kinase and the RR in bacteria suggest that multiple TCSs can be inhibited by a single compound, causing reduction in chromosomal resistance emergence (Barrett and Isaacson, 1995; Poole, 2012). As TCS-encoding genes are found in all Gram-positive and Gram-negative bacterial genomes, a particular polypharmacological TCS inhibitor with the desired therapeutic effect may act comprehensively, in contrast to polypharmacy practice, if able to breach the various cell envelopes (Anand and Chandra, 2014).

In this review, we list several TCSs that are linked to bacterial virulence and antibiotic resistance, pointing out some of their main characteristics and addressing their possible therapeutic uses as drug targets. Furthermore, the present review provides a compilation of TCS-inhibitors that have been identified by different assays of drug discovery.

## The Two-Component Systems and Their Involvement in Bacterial Virulence and Antibiotic Resistance

The basic biochemistry of the TCSs is well defined. A typical sensor histidine kinase comprises two domains: a variable N-terminal input domain and a conserved C-terminal domain that interacts with the RR. In turn, the RR contains one conserved N-terminal receiver domain and one variable C-terminal output domain (Nixon et al., 1986; Bijlsma and Groisman, 2003; Bourret and Silversmith, 2010; Cheung and Hendrickson, 2010; Groisman, 2016; Gislason et al., 2017). When stimulated, the sensor protein catalyzes autophosphorylation in a conserved histidine residue by using adenosine triphosphate (ATP). The high-energy phosphoryl group is then moved to a conserved aspartate residue of the RR, potentially changing its capability of targeting DNA sequences (Bijlsma and Groisman, 2003; Cheung and Hendrickson, 2010). This signal transduction pathway has been reported to involve three phosphotransfer reactions and two phosphoprotein intermediates (Stock et al., 2000; Bijlsma and Groisman, 2003).

In recent years, since protein phosphorylation has been discovered to take place in bacterial nitrogen absorption and chemotaxis, a number of techniques have been developed to study Two-component signal transduction systems (Scharf, 2010). The use of these techniques has allowed identification of stimuli-responsive TCSs, such as PhoPQ TCSs. *PhoPQ* is known to respond to a wide variety of environmental stress signals, including phosphate (Ogura et al., 2001; Pragai and Harwood, 2002), Mg^2+^ and Ca^2+^ starvation, pH, antimicrobial peptides and nutritional deprivation (Hall, 1998; Regelmann et al., 2002; Fontan et al., 2004; Miyashiro and Goulian, 2008). However, experimental verifications of stimuli that activate specific TCSs are reported in very few cases. When studying a TCS, a selection of candidate environmental conditions is usually made taking different physical and chemical parameters into consideration, such as changes in pH and osmolarity levels, oxygen pressure, temperature, exposure to ions, etc. (West and Stock, 2001; Varughese, 2002).

It has also been reported that some TCSs present the ability to regulate gene clusters that contribute to cell growth, biofilm formation and virulence in pathogenic bacteria (Eguchi and Utsumi, 2008; Mitrophanov and Groisman, 2008; Gotoh et al., 2010; Schaefers et al., 2017). Nevertheless, in several cases, the role of TCSs in the pathogenicity of bacteria is not well understood and the attenuation of virulence is observed in TCS mutant strains without immediate knowledge of the exact mechanisms involved. Even though there is little explanation about the mechanisms involved in their attenuation, the TCS mutant strains present great potential to be used as live-attenuated vaccines against bacterial infections.

For example, deletion of the genes encoding PhoP in *Mycobacterium* and *Salmonella* result in strains has been used as a vaccination strategy. The *phoP Salmonella* mutants are attenuated and immunogenic for virulence in animal models (VanCott et al., 1998; Link et al., 2006; Martin et al., 2006). Hohmann et al. (1996) demonstrated that the deletion of *phoP*/*phoQ* in *Salmonella typhi* provides a useful strain for immunogenic live-attenuated vaccine against typhoid fever. The association of TCSs with virulence has been studied in various pathogens, but few TCSs have been shown to be important for the coordination of expression of virulence factors. According to Sengupta et al. (2003), the *arcA* gene of *Vibrio cholerae* plays an essential role in the expression of virulence factors. The *arcA* gene was mutated and experimental animal infections revealed that this gene positively controls the expression of *toxT*. The gene *toxT* encodes a transcriptional factor that increases the virulence of *V. cholerae* by activating genes encoding CT and TcpA (Sengupta et al., 2003).

**FIGURE 1:**
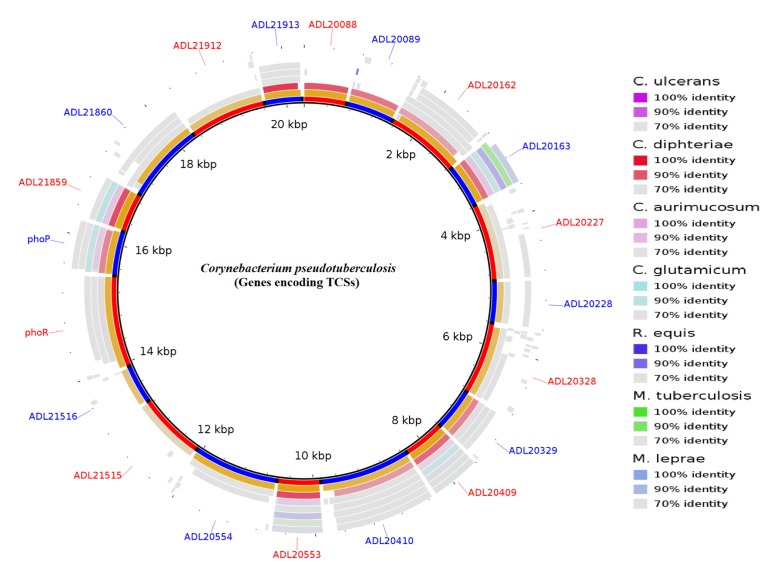
Circular representation of the genes encoding two-component systems (TCSs) in *Corynebacterium pseudotuberculosis*. The figure shows BLAST comparisons with other Actinobacteria.

The large number of available bacterial genome sequence databases has made it possible to identify and predict interacting pairs of response regulatory kinases. Among the approaches are the use of tools of Next-generation sequencing (NGS), molecular modeling, and bioinformatics. For example, our group is working with the genomics of pathogenic bacteria. *Corynebacterium pseudotuberculosis* (*Cp*) is the etiological agent of Caseous Lymphadenitis (CLA), an infectious disease that affects small ruminants worldwide (Dorella et al., 2006; Tiwari et al., 2014). The genome sequence of this bacterium, recently completed, will aid in the identification of homologies and the prediction of novel genes that encode other virulence factors. Genomic sequencing of *C. pseudotuberculosis* has revealed 10 of these signal transduction systems (**Figure 1**) (Barakat et al., 2011).

A number of research groups have recently focused on TCSs to find potent inhibitors. Examples of targeted TCSs are WalKR, which is essential for bacterial survival, QseCB and DosRST, both presenting an important role in virulence and VanSR, which exhibits antibiotic resistance properties. In fact, many envelope transporter proteins acting as efflux pumps to promote the secretion of toxic compounds are controlled by TCSs. It has been reported that the efflux pumps regulated by TCSs in several important human pathogens, including *Acinetobacter baumannii* and *Klebsiella pneumonia*, provide multidrug resistance (Howell et al., 2003; Gallego del Sol and Marina, 2013).

In specific, the VanSR TCS is responsible for regulating the genes that confer resistance to vancomycin. It has been reported that *Staphylococcus aureus, Enterococcus faecalis* and *E. faecium* are resistant to vancomycin because of the action of VanSR. Targeting of this TCSs has led to the identification of several potent inhibitors that arrest the energy required for ATP synthesis in the cell (Healy et al., 2000; Pootoolal et al., 2002; Hong et al., 2008; Bem et al., 2015). Due to the negative effect, they exert over the mitochondrial respiration, these inhibitors could not be readily used as drugs. However, they may serve as structural templates for the discovery of novel selective inhibitors to target the VanSR TCS in adjunct therapy.

Recently, in an attempt to modulate the activity of PhoPR TCS in *C. pseudotuberculosis*, our group evaluated potential binding interactions of prototype molecules from non-synthetic sources with the active site of the PhoP protein (Tiwari et al., 2014). As result, two anthraquinones produced by the plant species *Rheum undulatum* and *R. palmatum*, also known as rhein and reported to present antimicrobial activity, have been found to be good candidates to inhibit the activity of the PhoPR TCS in *C. pseudotuberculosis*.

Some predicted and experimentally validated compounds targeting bacterial TCSs are listed in **Table 1**. The firstly reported synthetic inhibitors target the *Pseudomonas aeruginosa* AlgR2/AlgRl TCS, which controls production of the exopolysaccharide alginate, an important virulence factor produced by this bacterium during lung infections (Roychoudhury et al., 1993). The principal approaches applied to the discovery of TCS inhibitors involve high throughput screening assays with purified TCSs and structure-based virtual screening (SBVS) using different types of compound libraries and rational drug design (Qin et al., 2006). By using these techniques, a number of synthetic compounds belonging to several classes, such as benzoxazines, benzimidazoles (Hilliard et al., 1998), bisphenols, cyclohexenes, trityls and salicylanilides (Hlasta et al., 1998), were identified to inhibit the KinA-Spo0F TCS in *Bacillus subtilis*. Concerning the effects of these compounds over bacterial growth, the 50% inhibitory concentrations (IC50s) ranged from 1.9 to 0.5 mM and the minimum inhibitory concentrations (MICs) ranged from 0.5 to 0.16 mg/ml for some bacteria (Hilliard et al., 1999; Stephenson and Hoch, 2002).

**Table 1:** Two-component systems targeted by molecular compounds with antibacterial activity.

Bacteria	Inhibitors	Two component system	Component of TCS studied	Mechanism of action	Reference
*Pseudomonas aeruginosa*	Thiazole derivatives	Algr1/Algr2	Sensor protein	Inhibition of phosphorylation/dephosphorylation of Algr2	Roychoudhury et al., 1993
*Enterococcus faecium*	Thiazole derivatives	VanR/VanS	Sensor protein	Inhibition of autophosphorylation	Ulijasz and Weisblum, 1999
*Bacillus subtilis*	Walkmycin B and Waldiomycin	WalK/WalR	Sensor protein	Binds to the HK cytoplasmic domain for the inhibition of autophosphorylation	Okada et al., 2010
*Staphylococcus aureus*	Walkmycin B and Waldiomycin	WalK/WalR	Sensor protein	Binds to the HK cytoplasmic domain for the inhibition of autophosphorylation	Okada et al., 2010
*Staphylococcus aureus*	Salicylanilide	KinA/Spo0F	Sensor protein	Affects membrane fluidity, disturbing signal transduction	Hilliard et al., 1999
*Bacillus subtilis*	Unsaturated fatty acids	KinA	Sensor protein	Causes non-competitive inhibition of ATP-dependent autophosphorylation	Barrett and Hoch, 1998
*Pseudomonas aeruginosa*	Thiazole derivatives	Algr1/Algr2	Response regulator	Inhibition of DNA-binding activity of Algr1	Roychoudhury et al., 1993
*Corynebacterium pseudotuberculosis*	Rhein	PhoP/PhoR	Response regulator	Inhibition of conserved receiver domain of PhoP	Tiwari et al., 2014
*Methicillin-resistant Staphylococcus aureus*	Bis-phenol	VanR/VanS	Response regulator	–	Barrett and Hoch, 1998
*Salmonella enterica*	NSC9608 (8 compounds, NCI library)	PhoP/PhoQ	Response regulator	Inhibition of formation of the PhoP-DNA complex	Tang et al., 2012


## Identification of Specific Inhibitors Against TCSs in Different Bacteria

Presently, several factors contribute to the urgent need for innovative therapeutics: (i) the rise of anti-microbial resistance in pathogenic bacterial strains worldwide, (ii) prolonged therapy, (iii) co-morbidity with immunosuppressive diseases like HIV, pooled with a rapid rate of microbial evolution, (iv) the bacterial latency caused when bacteria enter and reside in a dormant state even for several decades and (v) a slower development of new antibiotics. The world’s scientific community is facing a post-antibiotic era with a small number of available vaccines and a handful of chemotherapeutic agents available to battle infectious diseases (Worthington et al., 2013). In this regard, further basic research combined with modern drug discovery technologies may aid in the understanding of bacterial pathogenesis and cellular signaling pathways, leading to the development of novel therapeutic applications to exterminate bacterial infections.

Recognition of the relationship between TCSs and promotion of virulence in bacterial pathogens has directed the pharmaceutical industry to largely invest in the search of suitable inhibitors aiming signal transduction in bacteria. The catalytic and receiver domains of HKs and RRs are well studied and share a high degree of structural homology (Barrett and Hoch, 1998; Domagala et al., 1998). This knowledge suggests that a single drug compound capable of targeting any of these conserved domains could simultaneously inhibit multiple TCSs, boosting the chances to develop confrontation against the effects caused by the emergence of any molecular mutations affecting the affinity of the drug ligand for the target protein (Okada et al., 2007; Worthington et al., 2013). In this regard, targeting multiple TCSs through their conserved domains may be more effective than targeting the varying sensor domain of each HK (Gotoh et al., 2010; Bem et al., 2015). On the other hand, sequence conservation in kinase domains among bacteria and eukaryotes consists a possible disadvantage of targeting HKs. The ATP-binding pocket (Bergerat fold) present in bacterial HKs exhibits a high homology level with several human protein families and is also present in essential proteins found in a broad range of organisms, including the chaperone Hsp90 (Bem et al., 2015).

Computational programs relying on structure-activity relationship (SAR) have been used in medicinal chemistry studies aiming to identify compounds capable of inhibiting TCSs in pathogenic bacteria (Domagala et al., 1998; Weidner-Wells et al., 2001). Studies depicting the mechanisms of action of such compounds have revealed that some of the most promising TCS inhibitors – namely, the salicylanilides, trityl compounds and benzimidazoles – cause substantial hemolysis of equine erythrocytes, affect integrity of the cellular membrane in *S. aureus* and have a damaging effect on the synthesis of macromolecules (Hilliard et al., 1999; Stephenson and Hoch, 2002).

A number of halogenated pyrrolo [2, 1-b] [1, 3] benzoxazines (streptopyrroles) isolated from fermentations of *Streptomyces rimosus* have been shown to possess antimicrobial activity against a series of bacteria and fungi (Trew et al., 2000). The most promising streptopyrrole candidate presented inhibition against drug-resistant *S. aureus* strains at the MIC of 0.2 mg/mL. This candidate compound has also been able to repress auto-phosphorylation of the *Escherichia coli* NRII sensor kinase, providing an IC50 of 20 mM. (Stephenson and Hoch, 2002). However, similarities between the structures of streptopyrroles and salicylanilides suggest that the inhibition caused by these compounds may be a result of non-specific effects such as self-aggregation.

There are several methods to enhance the discovery/design of drugs targeting TCSs. First, the SBVS analysis should be performed using compound databases comprehending a wide variety of potential inhibitors, including structures reported to present antibacterial activity (Payne et al., 2007; Bem et al., 2015). Second, information about targeted TCSs should be obtained from clinically important pathogens to facilitate the identification of effective specific inhibitors. Third, the modeling refinement of TCS protein structures may facilitate the search for compounds using the SBVS approach by allowing a more precise molecular docking analysis. The structural information obtained from these proteins can also help to identify the binding sites and chemical spaces that allow the interaction with candidate compounds, leading to further refinement in the prediction of novel inhibitors. In this regard, structural data of proteins that have been co-crystalized with ligand molecules are of special interest. Finally, the generation of additional structure-activity relationship data will aid to minimize toxicity of the TCS inhibitors in eukaryotic cells and improve our understanding on the mechanisms of action of these compounds.

## Conclusion

The bacterial TCS consists of an important signaling mechanism for survival and establishment of pathogenic bacteria within the host. Along with the current need for new antimicrobial drugs, the regulatory nature of TCSs makes these systems outstanding targets for the development of alternative therapeutics against bacterial infections. Their broad prevalence and the functional diversity of TCSs also support the need for testing several compounds against TCSs. Although the structural conservation of domains present in histidine kinases and sensor proteins may facilitate the prediction of inhibitors, a substantial extent of similarity between bacterial TCSs and other eukaryotic related proteins provide important concerns in relation to the effectiveness of TCS-directed drugs, which could affect cellular processes in the host’s organism. In this regard, a better understanding of the interaction between a TCS and its targeting compound may aid in the development of an improved molecular structure presenting increased specificity for the correct ligand. Several works have reported natural and synthetic compounds presenting high affinity for TCSs and antimicrobial activity over pathogenic bacteria, but more studies are necessary to understand the exact mechanisms of action of these drugs.

## Author Contributions

ST, SBJ, and PVSDC wrote the paper. SSH, SA, AS, DB, TLPC, PG, and VA guided and reviewed the work.

## Conflict of Interest Statement

The authors declare that the research was conducted in the absence of any commercial or financial relationships that could be construed as a potential conflict of interest.
